# Symptomatic Bipartite Patella in Adults Treated With Open Excision: Outcomes and Management

**DOI:** 10.7759/cureus.26705

**Published:** 2022-07-09

**Authors:** Tommy Pan, William Hennrikus

**Affiliations:** 1 Orthopedic Surgery, Penn State College of Medicine, Hershey, USA; 2 Orthopedics, Penn State Health Milton S. Hershey Medical Center, Hershey, USA

**Keywords:** open excision, arthroscopy, sports medicine, bipartite patella, knee

## Abstract

Introduction

Bipartite patella affects about 2% of people. Most cases are asymptomatic; however, some develop anterior knee pain during a sporting activity or trauma. When conservative treatment fails, surgery can be considered. This study aims to report the outcomes of fragment excision with or without lateral release in adults with the symptomatic bipartite patella.

Methods

The study was approved by the College of Medicine IRB. A retrospective review was performed. Patients were excluded if aged < 18 or had prior knee surgery. Data collected included: age, gender, BMI, sports played, occupation, physical exam findings, Saupe classification, conservative and surgical treatment, advanced imaging used, duration of follow-up, Lysholm score and postoperative complications.

Results

Eight patients were studied. The average age was 28.4 years and BMI was 31.5. Sporting activities included hunting, swimming, soccer, golfing and softball. Occupations included office job, laborer, manufacturing plant worker and truck driver. All patients complained of anterior knee pain exacerbated by sports or work. All patients failed >6 months of conservative treatment. Saupe classification included seven types III (superolateral) and one type II (lateral). Surgical treatment included one open excision, six arthroscopic-assisted open excisions and one arthroscopic-assisted open excision with the lateral release. The duration of follow-up averaged 15 months. The average preoperative and postoperative Lysholm score was 75 and 93, respectively. One postoperative complication occurred.

Conclusions

Bipartite patella is an uncommon cause of anterior knee pain in adults. When pain persists despite conservative care, fragment excision of less than 12% of the whole patella with or without lateral release resulted in excellent outcomes in the majority of cases.

## Introduction

Bipartite patella (BP) is an anatomical variation found in 2% of the general population [1]. BP occurs when the secondary ossification centers of the patella fail to fuse during adolescence [2]. Starting at the age of three, the multiple ossification centers of the patella start to fuse from the center toward the periphery into the largest sesamoid bone of the human body [3]. However, failure of complete union results in the development of BP often connected by synchondrosis, a layer of fibrocartilage [4].

BP was first documented in 1883 by Wenzel Gruber in St. Petersburg, Russia, during the autopsy of a 21-year-old farmer who had a small piece of bone attached superolateral to the patella [3]. In 1921, Saupe classified BPa into three types in relation to the location of the accessory fragment with type I at the inferior pole (5%), type II at the lateral pole (20%) and, most commonly, type III at the superolateral pole (75%) [4].

In the majority of patients, BP is asymptomatic and often an incidental finding on radiographs; however, rarely, it can cause anterior patellofemoral knee pain and localized tenderness. Symptoms often start at adolescence during sporting activities. The majority of symptomatic BP can be successfully managed with conservative treatment including rest, stretching, brief immobilization, bracing, moderation of sporting activity, NSAIDs and physical therapy [5].

Current surgical modalities include open or arthroscopic excision, lateral retinacular release and open reduction internal fixation (ORIF) [2]. The purpose of this study is to report the outcomes of open fragment excision with or without lateral release in adults with the symptomatic BP.

## Materials and methods

**Figure 1:**
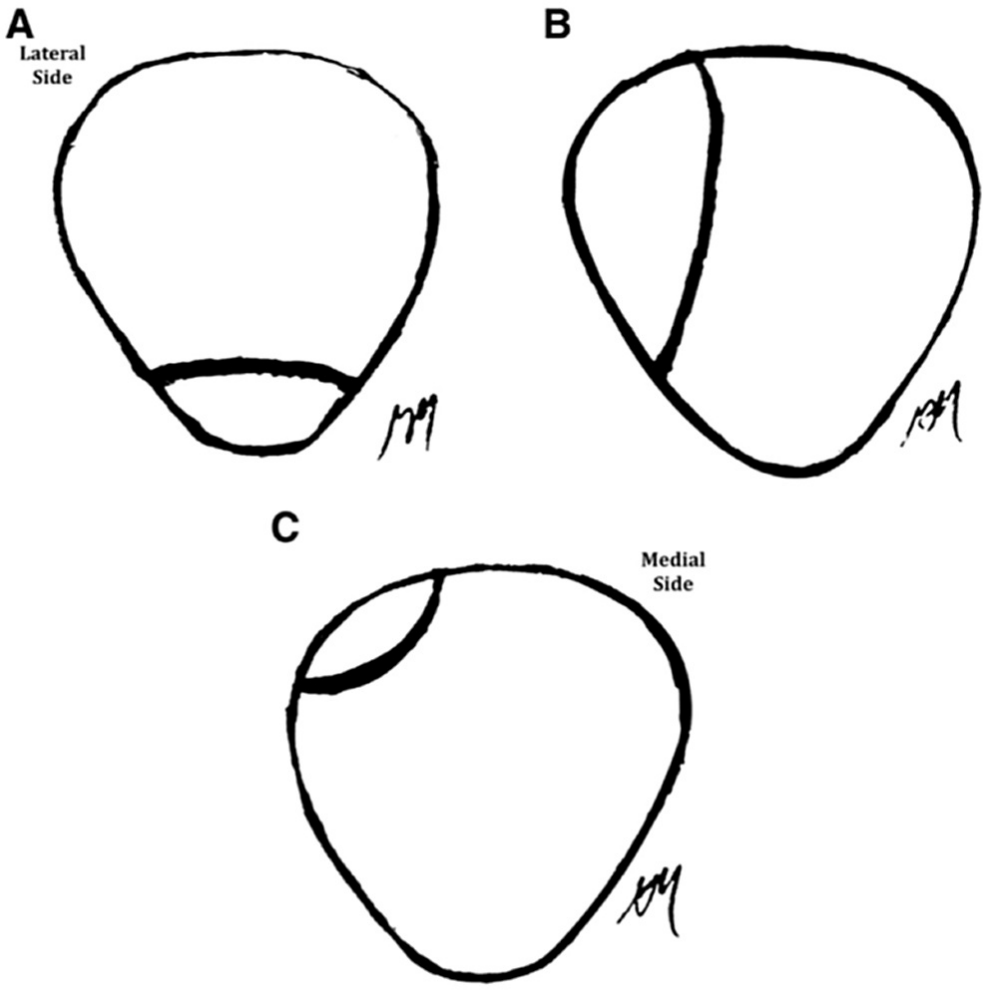
Saupe classification of the bipartite patella with regards to the location of the accessory fragment at the inferior (type I), lateral (type II) or superolateral (type III) margins of the patella.

The study was approved by the College of Medicine Institutional Review Board (IRB#:00012885). A retrospective chart and radiograph review were performed on patients who underwent surgery for symptomatic BP refractory to conservative treatment from 2009 to 2019. Patients who were excluded included: < 18 years of age or prior history of knee surgery. Data extracted included age, sex, laterality, body mass index (BMI), sporting activity, occupation, physical exam findings, conservative treatment, Saupe classification (Figure 1) [4], radiographic size of the fragment as a percentage of the entire patella, mechanism of injury (MOI), use of advanced imaging, surgical approach, duration of follow up, and complications. Clinical outcomes were measured using the Lysholm Scale [4]. The duration until return to full sporting activity and follow-up was recorded.

## Results

**Figure 2:**
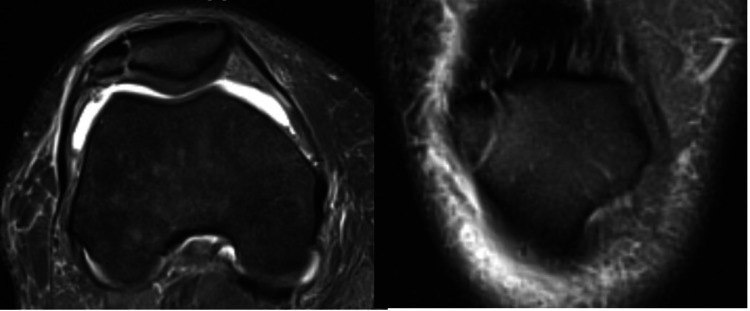
MRI with skyline (left) and AP (right) view of a bipartite patella in the lateral pole (Saupe Type II) in a 47-year-old male presenting with right anterior knee pain and tenderness. Case 6 (Table 1)

Eight patients and eight knees were studied (six males, two females). The average age was 28.4 years (range, 21 to 63 years) and BMI was 31.5 (range, 22 to 45). All patients complained of anterior knee pain exacerbated by sporting or work-related activities, especially with kneeling, twisting, squatting, jumping, or lifting heavy items. Sporting activities included hunting (two), swimming (one), soccer (one), golfing (one) and softball (one). The levels of sporting activity included seven recreational and one collegiate level. Occupations included manufacturing plant worker (two), truck driver (two), office job (one) and heavy-duty laborer (one). MOI included repetitive traction forces (three) and trauma (five), which were asymptomatic prior to the injury. On the physical exam, six had tenderness to palpation directly over the site of the bony division, five had mild effusion and one had crepitus at the patellofemoral joint. All knees demonstrated a full range of motion. All patients had failed >6 months of conservative treatment including PT, activity modification, stretching, bracing, steroid injections, Kinieseo taping, icing and NSAIDs (Table 1). Saupe classification included seven type III (superolateral) and one type II (lateral). One knee had lateral displacement and seven had normal positioning of the patella on patellofemoral tracking. The radiographic size of the fragment as a percentage of the overall size of the patella averaged 8.4% (range, 6% to 11%). This was obtained by dividing the surface area of the accessory fragment by the surface area of the patella on an AP radiographic view. Seven patients had advanced imaging with MRI or CT to rule out other intra-articular disorders (Figure 2). No additional intra-articular injuries were noted.

**Table 1:** Cohort general characteristics including age, gender, BMI, laterality, sporting activity, level of the sporting activity, Saupe classification, mechanism of injury, signs and symptoms and conservative management.

Case	Age	Gender	BMI	Laterality	Sporting Activity and/or Occupation	Level of Sporting Activity	Saupe Classification	Mechanism of Injury	Conservative Management	Signs and Symptoms
1	21	M	24	Left	Swimming	Recreational	III	Fracture after Fall at Home	Physical Therapy, Home Exercises, NSAIDs	Tenderness on Palpation, Lateral Patellar Pain
2	21	F	24	Left	Soccer	College	III	Repetitive Traction Forces	Activity Modification, Kinieseo Taping, Bracing, NSAID	Tenderness on Palpation, Mild Effusion
3	29	M	22	Left	Laborer, Heavy Duty Selector	Recreational	III	Car Accident Dashboard Injury	NSAIDs, Partial Weightbearing	Tenderness on Palpation, Mild Effusion
4	34	M	32	Left	Manufacturing Plant Worker	Recreational	III	Repetitive Traction Forces	NSAIDs, Partial Weightbearing, Crutches	Tenderness on Palpation, Mild Effusion
5	38	F	36	Right	Manufacturing Plant Worker, Softball	Recreational	III	Repetitive Traction Forces	Physical Therapy, Synvisc Injections, Bracing, NSAIDs	Tenderness on Palpation
6	47	M	45	Right	Truck Driver, Hunting	Recreational	III	Fall at Work	Physical Therapy, Knee Bracing, Crutches, NSAIDs	Tenderness on Palpation, Mild Effusion
7	49	M	36	Right	Truck Driver, Hunting	Recreational	III	Injury at Work	Physical Therapy, Synvisc Injections, Activity Modification, Bracing, NSAIDs	Tenderness on Palpation, Mild Effusion
8	63	M	33	Right	Office Job, Golfing	Recreational	II	Fracture after Fall at Home	Activity Modification, Icing	Tenderness on Palpation, Mild Crepitus

Surgical treatment included one open excision, six arthroscopic-assisted open excision and one arthroscopic-assisted open excision with lateral retinacular release (Table 2). Diagnostic arthroscopy was performed in seven knees to access the BP area, medial and lateral gutters and menisci, popliteus tendon attachment on the femur, fascicle attachments to the lateral meniscus, and medial patellofemoral ligament (MPFL). Chondral damage in these knees was graded using the International Cartilage Repair Society Grading System. Three knees had Grade I, three had Grade III and one had Grade IV chondral damage. There were no concomitant meniscal injuries. Postoperative management included: partial weightbearing as tolerated with crutches, knee bracing, icing, NSAIDs, physical therapy, a home exercise program and a gradual return to sporting activities or occupation at one month.

**Table 2:** Operative and postoperative findings including patellar dimension and surface area ratio, patellofemoral tracking, level of chondral damage, diagnosis with arthroscopy, surgical approach, Lysholm score and return to full sporting activity

Case	Dimensions of Accessory Fragment (mm)	Dimensions of Patella (mm)	Surface Area Ratio (%)	Patellofemoral Tracking	Level of Chondral Damage	Diagnosis w/ Arthroscopy	Surgical Approach	Lysholm Score	Return to Full Activity (Weeks)
1	21 x 13 x 14	60 x 50	9	Normal	Grade III	Yes	Open Excision	95	4
2	25 x 8 x 8	49 x 50	8	Normal	Grade I	Yes	Open Excision	98	4
3	20 x 11 x 11	48 x 51	9	Normal	Grade I	Yes	Open Excision	92	3
4	Not Recorded	Not Recorded	Not Recorded	Normal	Grade I	Yes	Open Excision	95	7
5	16 x 8 x 8	42 x 45	7	Normal	Grade III	Yes	Open Excision	90	Worker’s Comp
6	17 x 10 x 12	55 x 48	6	Lateral	Grade III	Yes	Open Excision, Lateral Release	93	4
7	14 x 12 x 10	40 x 46	9	Normal	Grade IV	Yes	Open Excision	95	6
8	27 x 12 x 12	54 x 56	11	Normal	Not Recorded	No	Open Excision	90	Worker’s Comp

The duration of follow-up averaged 15 months (range, 6-36 months). Compared to the average preoperative Lysholm score of 75 (range, 58-84), the average Lysholm Score at final follow-up was 93 (range, 90-98). Postoperatively, six patients returned to pre-surgery sporting or occupational activity at an average of 4.7 weeks (range, 4-7 weeks). Two patients received worker’s compensation. The most common postoperative finding was soft tissue swelling in five patients and 1 cm quadriceps muscle atrophy in four patients during the first-month follow-up visit. Thigh circumference was measured using a measuring tape and compared with that of the contralateral side. Two patients had chronic knee pain three years after surgery, one of whom applied for worker’s compensation.

One complication occurred during the follow-up period in a patient treated by open excision. A 38-year-old manufacturing plant worker developed a postoperative wound infection and was treated with antibiotics; she applied for worker’s compensation and did not return to work. None of the workers compensation patients had a secondary procedure within five years.

## Discussion

**Figure 3:**
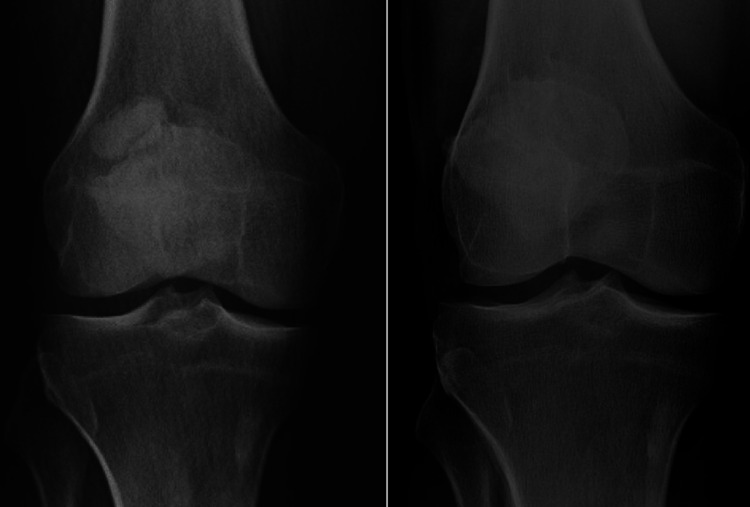
AP view of the preoperative (right) and postoperative (left) patellofemoral alignment in a 49-year-old male who underwent open excision with resection of the accessory fragment in the superolateral pole of the patella (Saupe Type III). Case 7 (Table 1)

BP is a rare cause of anterior knee pain due to the failure of the secondary ossification centers of the patella to completely fuse during adolescence. BP occurs in 2% of the population with equal prevalence in males compared to females. BP is often an incidental, asymptomatic finding on radiographs [2]. The etiology of the pain includes the repetitive traction forces of the extensor mechanism of the knee or the patellar maltracking caused by the “tight” lateral retinaculum pulling at the accessory fragment [3]. A direct blow to the knee synchondrosis, or vascular insufficiency, can also contribute to the pain [3,5]. BP is best visualized on plain radiographs in the anterior-posterior and “skyline” views (Figure 3) [5]. MRI can provide a valuable assessment of the morphological and pathological changes of BP, especially in determining the fragment height and distance between the fragment and patella [6,7].

The majority of symptomatic BP can be successfully treated conservatively with activity modification, NSAIDs, icing, physical therapy, bracing or ultrasound-guided injections [8]. When conservative treatment fails, surgery can then be considered [9]. Few studies have reported on the outcomes of operative treatment in the adult population [5,10,11].

Open excision with resection of the accessory fragment is the most commonly performed procedure in adults [12,13]. Lateral release may reduce excess lateral traction force, denervate the pain fibers to the fragment, improve the patellofemoral tracking and promote bony union [13-16]. In the current study, open fragment excision with or without lateral release on eight knees yielded four (50%) good and four (50%) excellent results. The decision to perform the lateral release was made on a case-by-case basis by the individual surgeon. An overdone lateral release can result in medial patella instability. No case of medial instability occurred in the current paper.

When opting for excision with resection of the accessory fragment, it is important to consider the percentage of the patella that can be safely resected to minimize patellar instability, joint incongruity and arthritis [17]. No more than 40% of the patella should be removed [18]. Excessive excision can decrease the mechanical advantage that the patella provides to the extensor mechanism [19]. In the study with excision, an average of 7.4% (range, 6% to 11%) of the patella was resected.

Other operative modalities of symptomatic BP include arthroscopic excision, tension band wiring or ORIF [7,20]. In arthroscopic excision, the anteromedial and anterolateral portals provide excellent access to the BP fragment with the knee in full extension [21,22]. Compared to open excision, arthroscopic excision provides faster recovery, fewer complications, less pain, and expedited return of muscle strength, knee effusion resolution, and return to work or sport [3,23]. Further, arthroscopy offers advantages both for diagnostic recognition and intraoperative treatment. The long-term outcomes of the two techniques, however, are similar.

Tauber et al. reported an 18-year-old who suffered a traumatic separation from the synchondrosis while playing soccer. He was treated with tension band wiring of the atypical, large, and horizontal BP resulting in a successful bony union of the BP [24]. Vaishya et al. reported ORIF of the BP using a compression screw in two patients with fragments measuring 2.66 and 2.61 cm^2^, respectively. Excision of the large fragments in these cases may have led to the incongruity of the patellofemoral joint and increased risk of arthritis [25]. ORIF with extensor mechanism repair provides successful outcomes in reported cases of traumatic separation of BP with concomitant quadriceps tendon rupture [20]. In 2015, Matic et al. evaluated the return to activity in athletes with symptomatic BP treated by excision, lateral release, or ORIF. Fragment excision was associated with the best results, with a 91% rate of symptom relief and return to full sporting activity [25].

Limitations of this study include: 1) the small number of cases investigated due to the rarity of this pathological finding; 2) the lack of a control group and 3) the limited period of follow-up. Given the low prevalence of this condition in the normal population that is operated on, a randomized control was not possible.

The current study reaffirms the existing literature. Open excision with or without lateral release followed by early patella, quadriceps, and patellar tendon mobilization serves as a viable surgical modality in treating adults with symptomatic BP. Excision of the accessory fragment, averaging approximately 7% of the overall patella, relieves pain and preserves the patellar integrity. Lateral release reduced the painful tracking forces applied to the synchondrosis. Postoperative outcome scores were good to excellent using the Lysholm scale at an average of 15 months’ follow-up. Complete return to sporting activity or work is patient-dependent and individualized according to the patient's discretion.

## Conclusions

Symptomatic BP is an uncommon cause of anterior knee pain in adults. Open excision with or without lateral release followed by early patella, quadriceps, and patellar tendon mobilization serves as a viable surgical modality in treating adults with symptomatic BP. When pain persists despite conservative care, excision of the accessory fragment of less than 12% of the overall patella relieves pain and preserves the patellar integrity. Lateral release reduced the painful tracking forces applied to the synchondrosis. Postoperative outcome scores were good to excellent with excellent pain relief and return to full sporting or occupational activity in the majority of cases.
